# Alcoholic liver disease confers a worse prognosis than HCV infection and non-alcoholic fatty liver disease among patients with cirrhosis: An observational study

**DOI:** 10.1371/journal.pone.0186715

**Published:** 2017-10-27

**Authors:** Astrid Marot, Jean Henrion, Jean-François Knebel, Christophe Moreno, Pierre Deltenre

**Affiliations:** 1 Division of Gastroenterology and Hepatology, Centre Hospitalier Universitaire Vaudois, Université de Lausanne, Lausanne, Switzerland; 2 Division of Gastroenterology and Hepatology, Centres hospitaliers de Jolimont, Haine-Saint-Paul, Belgium; 3 Laboratory for Investigative Neurophysiology (The LINE), Department of Radiology and Department of Clinical Neurosciences, University Hospital Center and University of Lausanne, Lausanne, Switzerland; 4 EEG Brain Mapping Core, Centre for Biomedical Imaging (CIBM), Lausanne, Switzerland; 5 Department of Gastroenterology, Hepatopancreatology and Digestive Oncology, CUB Hôpital Erasme, Université Libre de Bruxelles, Brussels, Belgium; Medizinische Fakultat der RWTH Aachen, GERMANY

## Abstract

**Background:**

Cirrhosis is a heterogeneous clinical condition that includes patients at wide-ranging stages of severity. The role of the underlying liver disease on patient prognosis remains unclear.

**Aim:**

To assess the impact of the underlying liver disease on the occurrence of hepatocellular carcinoma (HCC) and death.

**Methods:**

Data related to the occurrence of HCC and death were collected during a 21-year period among patients with cirrhosis related to alcoholic liver disease (ALD) (n = 529), chronic hepatitis C virus (HCV) infection (n = 145) or non-alcoholic fatty liver disease (NAFLD) (n = 78).

**Results:**

At inclusion, ALD patients were younger than HCV and NAFLD patients (56 vs. 67 vs. 63 years; p<0.001) and had worse liver function (percent of patients with Child-Pugh stages B or C: 48% vs. 8% vs. 17%; p<0.001). During follow-up, 85 patients developed HCC and 379 died. The 10-year cumulative incidence rate of HCC was lower in ALD patients than in HCV and NAFLD patients (8.4% vs. 22.0% vs. 23.7%; p<0.001). The 10-year cumulative incidence rates of mortality were not statistically different between ALD, HCV and NAFLD patients (58.1% vs. 47.7% vs. 49.9%; p = 0.078). Alcohol abstinence and viral eradication were associated with reduced mortality among ALD and HCV patients, respectively. In multivariate analyses, ALD was associated with a reduced risk of HCC (0.39; 95% CI, 0.20–0.76; p = 0.005) but with a higher risk of mortality (1.53; 95% CI, 1.20–1.95; p<0.001). ALD patients died more frequently from decompensation of cirrhosis.

**Conclusion:**

Despite a lower incidence of HCC, patients with ALD-related cirrhosis have a worse outcome than those with chronic HCV infection or NAFLD-related cirrhosis.

## Introduction

Cirrhosis is associated with high mortality rates, and is the main indication for liver transplantation. It is a heterogeneous clinical condition that includes patients with different causes of chronic liver disease and at wide-ranging stages of severity. Hence, precise long-term prognosis assessment for these patients is required. Although several studies have provided data on the natural history of alcoholic liver disease (ALD), chronic hepatitis C virus (HCV) infection and nonalcoholic fatty liver disease (NAFLD), the outcome of cirrhosis according to the cause of the disease has been poorly investigated. More particularly, current available data regarding the overall prognosis as well as the risk of hepatocellular carcinoma (HCC) among patients with ALD-related cirrhosis are controversial. A poor survival has been reported among cirrhotic patients with ALD compared to those with HCV infection [1] while other studies have indicated that the cause of liver disease does not affect prognosis [2–5]. In addition, chronic HCV infection may confer a greater risk of HCC than ALD or NAFLD [3, 4, 6–8]. Overall, the prognosis of patients with ALD-related cirrhosis remains poorly defined and further studies assessing whether ALD confers a different prognosis among patients with cirrhosis than chronic HCV infection or NAFLD are essential to improve patient care.

Accurate analysis of cirrhotic patient’s outcomes requires a large sample of individuals followed for a long period of time and the use of appropriate models of disease progression. For this purpose, we sought to assess the development of HCC as well as all-cause of mortality and liver-related mortality among patients with cirrhosis according to the etiology of liver disease using competing risk analysis. We followed a large series of patients with cirrhosis due to ALD, chronic HCV infection or NAFLD consecutively seen in a single center during a 21-year period and we collected data related to the occurrence of HCC and death.

## Materials and methods

### Patients

Between January 1995 to December 2014, all patients referred from the outpatient clinic at Jolimont Hospital were consecutively enrolled by one of us in a registry if they fulfilled the following criteria: (1) age >18 years; (2) cirrhosis demonstrated by liver biopsy showing fibrotic nodules consistent with a METAVIR F4 fibrosis stage or by unequivocal signs of cirrhosis (dysmorphic liver, ascites, esophageal or gastric varices); (3) cirrhosis related to ALD, chronic HCV infection or NAFLD. All stages of cirrhosis were included. Patients with cirrhosis unrelated to one of these causes were excluded, as well as patients who developed HCC or who died within 3 months of their inclusion in the registry. HCV patients with excessive alcohol intake (more than 210 g/week for men and more than 140 g/week for women) were also excluded. During follow-up, patients were followed as outpatients every six-months, or more frequently if required. Data related to the development of HCC, to the occurrence of liver transplantation or death were collected. Examination by Doppler ultrasonography was performed every 6 months for HCC surveillance. When this registry began, written informed consent and ethical committee approval were not mandatory. As this registry has not been granted ethics committee approval prior to commencing, retrospective ethics approval cannot be obtained. However, patients were informed about their participation to an observational registry as soon as it became a rule for an observatory study to ask patient’s consent and patients gave informed consent verbally.

### Data collection

Baseline data and follow-up data related to the occurrence of HCC, death and causes of death were recorded. For patients lost to follow-up, patient charts were retrospectively reviewed, and in case of missing data, patients’ family and/or their general practitioner were contacted to know if the patient was alive and, in case of death to obtain information on time and cause of death. The last data were collected on 31^st^ December 2015.

### Etiology of liver disease

Patients were divided into 3 groups according to the cause of cirrhosis: ALD, chronic HCV infection or NAFLD. The determination of the main cause of cirrhosis was made at inclusion in the registry using a set of clinical, biological, morphological and histological data. Most of these data were confirmed on multiple occasions during follow-up.

The diagnosis of ALD was based on the following criteria: (1) history of excessive chronic alcohol intake greater than 210 g/week for men and greater than 140 g/week for women during several years; (2) absence of another obvious cause of cirrhosis. The diagnosis of chronic HCV infection was based on the following criteria: (1) detection of anti-HCV; (2) positivity for HCV RNA; (3) absence of excessive alcohol consumption (less than 210 g/week for men and less than 140 g/week for women); (4) absence of another obvious cause of cirrhosis. Anti-HCV antibodies were determined using a chemiluminescence immunoassay (Architect^®^ anti-HCV assay; Abbott Diagnostics, Abbott Park, IL, USA). Levels of serum HCV RNA were quantified by real-time PCR. Two standardized PCR tests were used for HCV RNA detection and quantification: COBAS AMPLICOR^™^ Hepatitis C Virus (HCV) Test, version 2.0 (v2.0) with a detection and quantification limit of 50 IU/ml (before 2008), or Abbott RealTime HCV with a detection and quantification limit of 12 IU/ml (after 2008). The diagnosis of NAFLD was based on the following criteria: (1) absence of excessive alcohol consumption (less than 210 g/week for men and less than 140 g/week for women); (2) absence of another obvious cause of cirrhosis; (3) when liver biopsy was performed, presence of histological features of NAFLD. The diagnosis of NAFLD was also considered in case of cryptogenic cirrhosis with features of metabolic syndrome such as obesity and/or diabetes [9, 10].

### Study outcomes

The main end-points were the development of HCC and death. Diagnosis of HCC was made by non-invasive radiological criteria using contrast-enhanced imaging techniques showing contrast uptake in the arterial phase and washout in the venous phase according to guidelines at that time (in most cases, one imaging technique for nodules >2 cm and two coincidental techniques for nodules 1–2 cm in diameter) or by histological examination. Deaths due to HCC or decompensation of cirrhosis (either acute-on-chronic liver failure or end-stage liver disease) were considered liver-related. All other causes of deaths were considered non liver-related.

### Statistical analysis

Data were expressed as percentage or median (95% CI). In a first step, an overall analysis was made. In a second step, a subgroup analysis in Child-Pugh A patients was performed. Analyses were conducted using variance analysis, the chi-square test, two-sided Fisher exact test, Mann-Whitney test, Wilcoxon test and two-sample Student’s t-test when appropriate. Follow-up started at the inclusion of patients. Data of patients who did not die were censored at the time of last contact or on December 31^th^ 2015, whichever was earlier. Time-to-event was calculated from the date of inclusion to the date of first detection of HCC or death. We used cumulative incidence functions to describe the probability of an endpoint at a given time, as recommended [11]. The risk of HCC was described with the cumulative incidence function taking into account death from liver decompensation, death from non liver-related causes and liver transplantation as competing risks. The risk of death was estimated with the cumulative incidence function taking into account liver transplantation as a competing risk. The risk of liver-related death was estimated with the cumulative incidence function taking into account death from non liver-related causes and liver transplantation as competing risks. The Gray’s test was used to test the causes-specific death differences [12]. Average annual rates were estimated as numbers of events divided by the number of person-year at-risk. All results were reported with their 95% confidence interval (95% CI).

We conducted univariate and multivariate Fine and Gray proportional hazards models to identify factors associated with HCC or death. Covariates included in the multivariable model were age, gender and etiology of cirrhosis. Then, we calculated Akaike information criterion (AIC) values by adding other variables using a forward step by step approach [13–15]. Variables available in less than 75% of the patients were not considered in multivariate analyses. To avoid bias related to the effect of colinearity, when Child-Pugh or MELD scores were included in multivariate analysis, their constituent variables were not considered. Hazard ratios (HR) were reported with 95% CIs. All tests were two-tailed and a *p-Value* of less than 0.05 was considered to be statistically significant.

In patients with cirrhosis related to ALD, the impact of abstinence on patient prognosis was assessed. Abstinence was defined as discontinuation of any alcohol intake within the first 12 months following the first visit. In patients with cirrhosis related to HCV infection who received antiviral treatment, we assessed the impact of viral eradication on the occurrence of HCC, death and liver-related death as follows. First: viral eradication was considered to be a variable that could change over time. For patients receiving antiviral treatment and reaching sustained virological response (SVR, defined by undetectable HCV-RNA 24 weeks after the end of treatment), observation time was censored when successful antiviral treatment was ended. This choice was justified by the fact that SVR patients were all HCV RNA–negative at the end of a successful antiviral treatment. Patients having reached SVR were considered as non-SVR patients until the end of the successful treatment, and thereafter as SVR patients until the end of the follow-up. Second: the association between viral eradication and each endpoint was tested in univariate and multivariate analyses.

Univariate analyses were performed using NCSS 2007 software (NCSS, Kaysville, UT, USA). Fine and Gray proportional hazards models, cumulative incidence function and AIC stepwise procedure were analyzed using Anaconda 2.7 a free distribution of the Python programming language (Python Software Foundation. Python Language Reference, version 2.7.), the python module Rpy2 (Available at https://pypi.python.org/pypi/rpy2) to link python with R 3.1.3 (R Core Team (2015). R: A language and environment for statistical computing. R Foundation for Statistical Computing, Vienna, Austria.URL http://www.R-project.org/), the R libraries “cmprsk” (Bob Gray (2014). cmprsk: Subdistribution Analysis of Competing Risks. R package version 2.2–7. http://CRAN.R-project.org/package=cmprsk) and “crrstep” (Ravi Varadhan & Deborah Kuk (2015). crrstep: Stepwise Covariate Selection for the Fine & Gray Competing Risks Regression Model. R package version 2015–2.1. http://CRAN.R-project.org/package=crrstep).

## Results

### Study population

**Fig 1:**
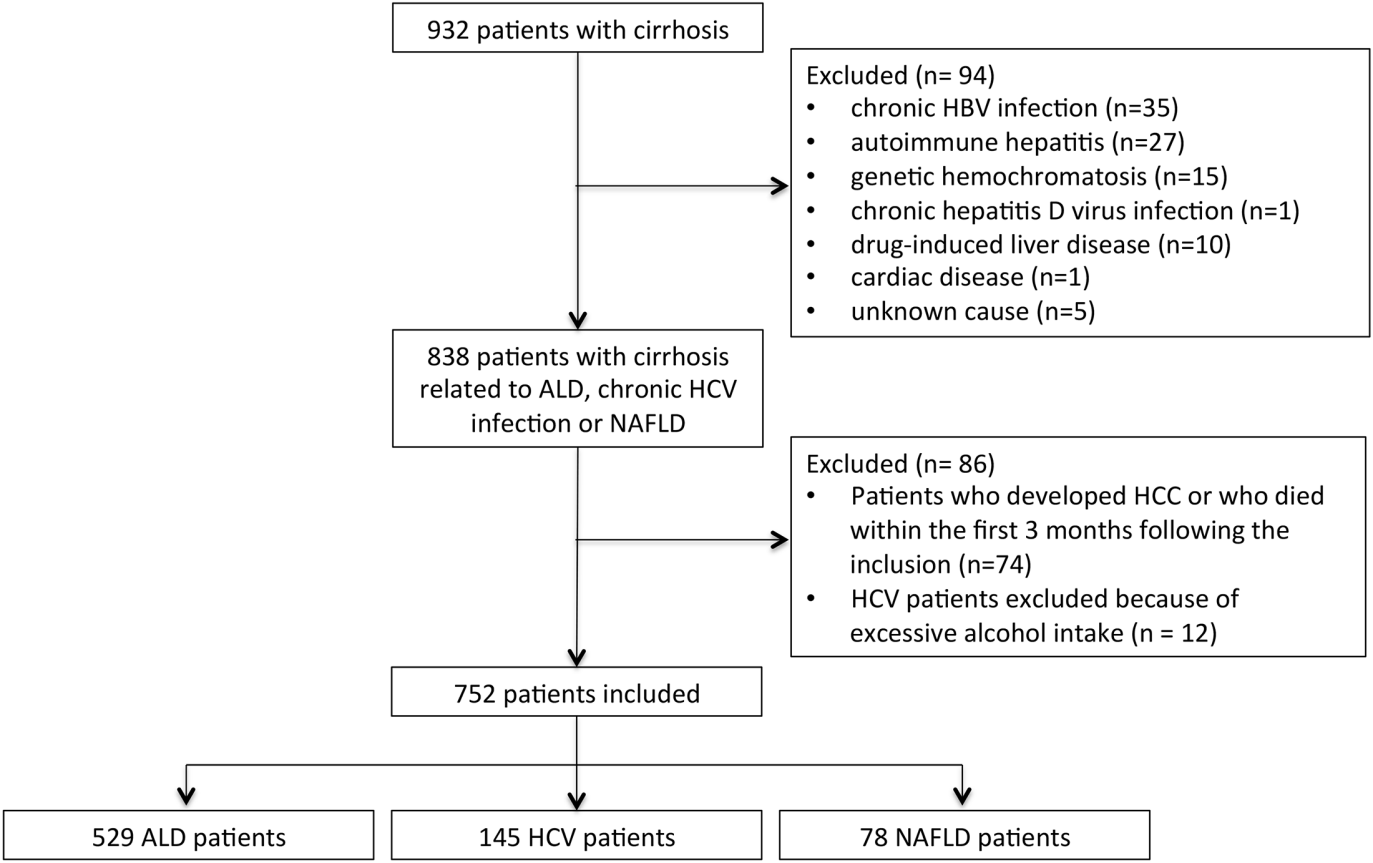
Flowchart of the study population. ALD, alcoholic liver disease; HBV, hepatitis B virus; HCC, hepatocellular carcinoma; HCV, hepatitis C virus; NAFLD, non-alcoholic fatty liver disease.

Fig 1 presents the flowchart of the study population. From January 1995 to December 2014, 932 patients with cirrhosis were identified. Of these patients, 94 were excluded because they had cirrhosis related to chronic hepatitis B virus infection (n = 35), autoimmune hepatitis (n = 27), genetic hemochromatosis (n = 15), chronic hepatitis D virus infection (n = 1), drug-induced liver disease (n = 10), cardiac disease (n = 1) or cirrhosis of an unknown cause (n = 5). In addition, 74 patients were excluded because they died or suffered from HCC within the first 3 months following their inclusion. Lastly, 12 HCV patients were excluded because of excessive alcohol intake. Thus, 752 patients were included. There were 529 (70%) patients with ALD-related cirrhosis, 145 (19%) with HCV-related cirrhosis, and 78 (10%) with NAFLD-related cirrhosis. The diagnosis of cirrhosis was made by liver biopsy in 418 patients (56%). The median time from inclusion to last available data was 58 months (95% CI: 52–63, 55 months among ALD patients, 91 months among HCV patients and 50 months among NAFLD patients, p<0.001). 14 patients were lost to follow-up with no available data related to death, causes of deaths or liver transplantation.

### Baseline data

Baseline patient’s characteristics are shown in Table 1. The median age was 57 years (95% CI: 57–58) and 479 patients were male (64%). When compared to HCV and NAFLD patients, ALD patients were younger (56 vs. 67 vs. 63 years; p<0.001) and had worse liver function (percent of patients with Child-Pugh stages B or C: 48% vs. 8% vs. 17%, respectively; p<0.001).

**Table 1:** Characteristics of the study population at inclusion according to the cause of cirrhosis.

Characteristics	Whole population (n = 752)	ALD group (n = 529)	HCV group (n = 145)	NAFLD group (n = 78)	*p-Value*
Age (years) *	57 (57–58)	56 (55–56)	67 (64–69)	63 (58–67)	< 0.001
Male sex (no of males, %)	479 (64%)	362 (68%)	77 (53%)	40 (51%)	< 0.001
Diabetes (no, %)	178 (39%) **	106 (34%)	22 (29%)	50 (73%)	< 0.001
Tobacco use (no of consumers, %)	178 (62%) ***	143 (66%)	15 (42%)	20 (57%)	0.02
BMI (kg/m^2^) *	28 (27–29) ****	26 (25–28)	27 (24–29)	31 (30–32)	< 0.001
Bilirubin levels (mg/dl) *	1.1 (0.9–1.2)	1.3 (1.2–1.5)	0.8 (0.7–0.8)	0.8 (0.6–0.9)	< 0.001
INR *	1.1 (1.1–1.1)	1.1 (1.1–1.1)	1.0 (1.0–1.0)	1.0 (1.0–1.1)	< 0.001
Albumin levels (g/dl) *	3.9 (3.8–4.0)	3.7 (3.5–3.8)	4.1 (4.0–4.2)	4.2 (4.1–4.4)	< 0.001
Creatinin levels (mg/dl) *	0.8 (0.8–0.9)	0.8 (0.8–0.8)	0.9 (0.8–0.9)	0.9 (0.8–1.0)	< 0.001
Platelet count (10^3^/mm^3^) *	141 (134–150)	141 (132–152)	127 (116–140)	165 (141–180)	0.004
Presence of esophageal or gastric varices (no, %)	230 (53%) °	189 (58%)	19 (35%)	22 (41%)	0.002
Child-Pugh score *	5 (5–6)	6 (6–7)	5 (5–5)	5 (5–5)	< 0.001
Child-Pugh classification					< 0.001
Child-Pugh class A (no, %)	473 (63%)	275 (52%)	133 (92%)	65 (83%)	
Child-Pugh class B (no, %)	194 (26%)	169 (32%)	12 (8%)	13 (17%)	
Child-Pugh class C (no, %)	85 (11%)	85 (16%)	0 (0%)	0 (0%)	
MELD score *	8.4 (8.0–8.8) °°	9.2 (8.5–9.9)	7.3 (6.5–7.5)	7.5 (6.4–8.4)	< 0.001

Abbreviations: ALD, alcoholic liver disease; CI, confidence interval; HCC, hepatocellular carcinoma; HCV, hepatitis C virus; NAFLD, non-alcoholic fatty liver disease

* Data are expressed as the median (95% CI)

** Data available in 459 patients

*** Data available in 289 patients

**** Data available in 259 patients

° Data available in 436 patients

°° Data available in 478 patients

### HCC, deaths, causes of death and liver transplantation

Data related to HCC, death and liver transplantation occurring during the study period are reported in S1 Table. Eighty-five patients developed HCC (11%). Among these patients, 38 had ALD-related cirrhosis (7% of all ALD patients), 35 had HCV-related cirrhosis (24% of all HCV patients) and 12 had NAFLD-related cirrhosis (15% of all NAFLD patients) (p<0.001). HCC was diagnosed during HCC surveillance with Doppler ultrasonography examination performed every 6 months in 65 cases (26 ALD patients, 28 HCV patients and 11 NAFLD patients, p = 0.2) and outside the 6 months surveillance program in 20 cases. HCC was diagnosed within Milan criteria [16] in 68 cases (28 ALD patients, 28 HCV patients and 12 NAFLD patients, p = 0.14).

Three hundred and seventy-nine patients (51% of the whole study population) died during the follow-up. Causes of death were liver-related in 250 patients (66%), non-liver related in 123 patients (32%) and unknown in 6 patients (2%). Among the non liver-related deaths, 39 were related to extra-hepatic malignancies, 7 to strokes, 26 to cardiovascular diseases, 12 to advanced pulmonary diseases, 13 to accidental deaths and 26 to other causes. Among the 379 patients who died, there were 273 ALD patients (53% of all ALD patients), 78 HCV patients (55% of all HCV patients) and 28 NAFLD patients (36% of all NAFLD patients) (p = 0.01). Among ALD patients, cause of death was HCC in 12 cases (5% of deceased patients), decompensation of cirrhosis in 178 cases (65%), and non liver-related in 80 cases (29%). Among HCV patients, causes of death were HCC in 21 cases (27% of deceased patients), decompensation of cirrhosis in 25 cases (32%), and non liver-related in 30 cases (38%). Among NAFLD patients, causes of death were HCC in 4 cases (14% of deceased patients), decompensation of cirrhosis in 10 cases (36%), and non liver-related in 13 cases (46%) (p<0.001). Age at death was lower for ALD patients than for HCV or NAFLD patients (61 vs. 76 vs. 77 years, respectively; p<0.001).

Twenty-seven patients (4% of the whole population) underwent a liver transplantation during the study period, 17 ALD patients, 8 HCV patients and 2 NAFLD patients.

### Cumulative incidence of HCC and factors predicting HCC

**Fig 2:**
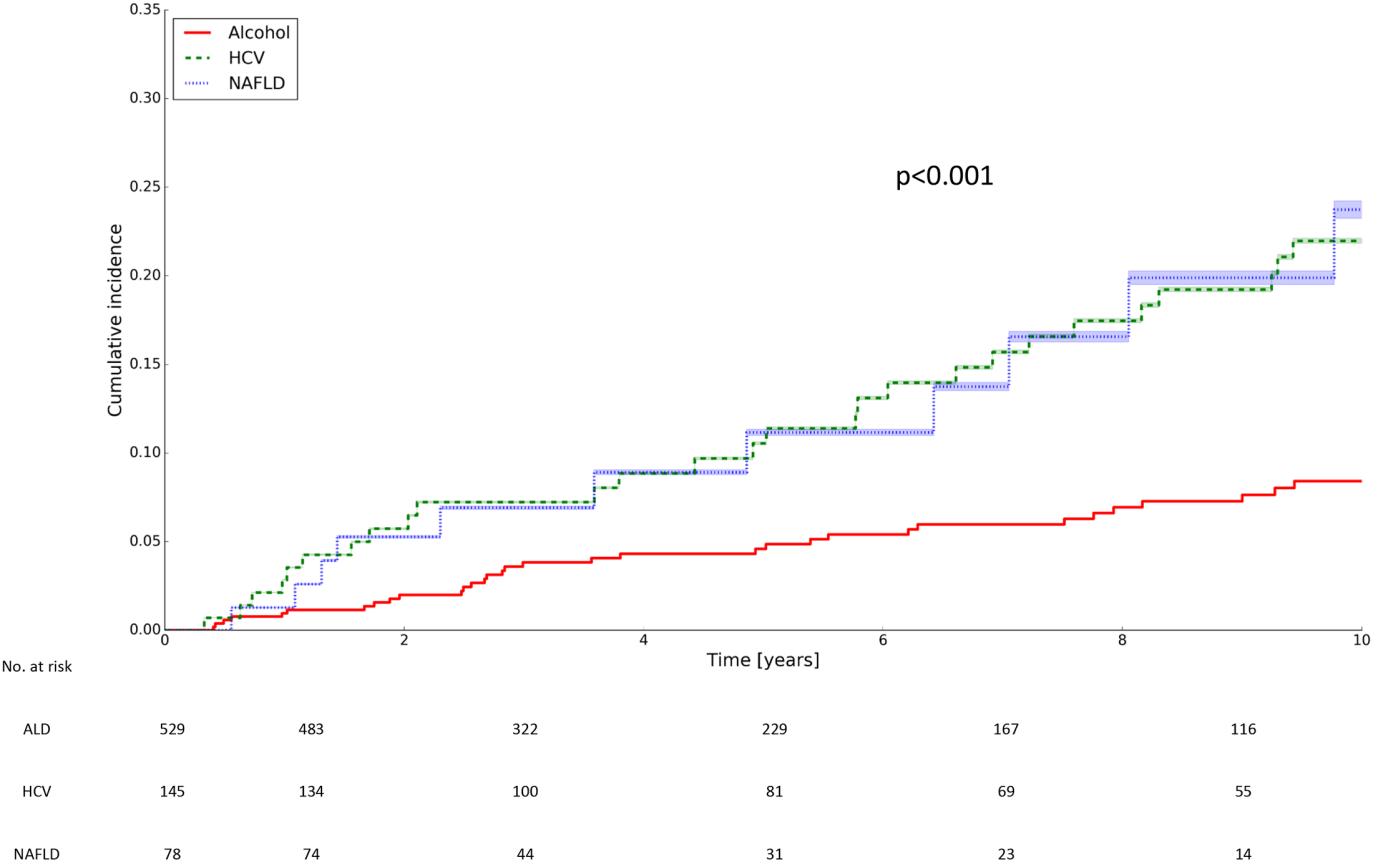
Cumulative incidence rate of HCC according to the cause of cirrhosis. ALD, alcoholic liver disease; HCC, hepatocellular carcinoma; HCV, hepatitis C virus; NAFLD, non-alcoholic fatty liver disease.

S2 Table provides the 5 and 10-year cumulative incidence rates of HCC in patients with ALD, HCV and NAFLD-related cirrhosis. The 10-year cumulative incidence rates of HCC were significantly lower in ALD patients than in HCV or NAFLD patients (8.4% vs. 22.0% vs. 23.7%, respectively, p<0.001; Fig 2). The annual cumulative risks of HCC were 1.1%, 2.9% and 3.1% in ALD, HCV and NAFLD patients, respectively.

In time-dependent multivariate proportional hazards models, factors associated with the development of HCC were older age (HR: 1.03; 95% CI, 1.00–1.06; p = 0.03), gender (HR for male gender: 2.41; 95% CI, 1.39–4.15; p = 0.002) and etiology of cirrhosis (HR for ALD: 0.39; 95% CI, 0.20–0.76; p = 0.005) (Table 2). In subgroup analysis restricted to Child-Pugh A patients (n = 473), factors associated with the development of HCC were gender (HR for male gender: 3.57; 95% CI, 1.83–6.96; p<0.001), the presence of diabetes (HR: 1.77, 95% CI: 1.00–3.15, p = 0.05), and etiology of cirrhosis (HR for ALD: 0.37; 95% CI, 0.18–0.77; p = 0.008) (S3 Table).

**Table 2:** Risk factors for HCC and for death.

		HCC				Mortality				Liver-related mortality			
		Univariate		Multivariate		Univariate		Multivariate		Univariate		Multivariate	
Baseline characteristics	Comparison group	Hazard ratio (95% CI)	*p-Value*	Hazard ratio (95% CI)	*p-Value*	Hazard ratio (95% CI)	*p-Value*	Hazard ratio (95% CI)	*p-Value*	Hazard ratio (95% CI)	*p-Value*	Hazard ratio (95% CI)	*p-Value*
Age	1-year increase	1.04 (1.03–1.06)	< 0.001	1.03 (1.00–1.06)	0.03	1.03 (1.02–1.04)	< 0.001	1.05 (1.04–1.06)	< 0.001	1.01 (1.00–1.02)	0.01	1.03 (1.02–1.04)	< 0.001
Gender	Male vs. female	1.67 (1.04–2.67)	0.03	2.41 (1.39–4.15)	0.002	1.24 (1.01–1.53)	0.04	1.36 (1.10–1.69)	0.005	1.25 (0.97–1.61)	0.09	1.38 (1.05–1.80)	0.02
Etiology of cirrhosis	ALD vs. non ALD	0.33 (0.21–0.50)	< 0.001	0.39 (0.20–0.76)	0.005	1.28 (1.03–1.60)	0.03	1.53 (1.20–1.95)	< 0.001	1.55 (1.16–2.06)	0.003	1.38 (1.00–1.91)	0.05
Diabetes	Yes vs. no	1.36 (0.84–2.20)	0.2	1.48 (0.86–2.53)	0.15	1.18 (0.87–1.59)	0.3			0.99 (0.69–1.41)	0.9		
Tobacco use	Yes vs. no	1.49 (0.76–2.94)	0.2			0.88 (0.60–1.27)	0.5			0.76 (0.49–1.19)	0.2		
BMI	1-point increase	1.04 (1.00–1.09)	0.06			0.97 (0.93–1.00)	0.09			0.99 (0.95–1.04)	0.8		
Bilirubin	1 mg/dl increase	1.00 (0.91–1.11)	0.9			1.07 (1.01–1.13)	0.03			1.10 (1.03–1.16)	0.002		
INR	1-point increase	0.83 (0.21–3.30)	0.8			3.47 (1.86–6.50)	< 0.001			5.42 (2.82–10.44)	< 0.001		
Albumin	1 g/dl increase	1.02 (0.75–1.39)	0.9			0.62 (0.50–0.75)	< 0.001			0.58 (0.46–0.73)	< 0.001		
Creatinin	1 mg/dl increase	1.57 (0.67–3.66)	0.3			2.66 (1.46–4.85)	0.001			2.09 (0.98–4.45)	0.06		
Platelet count	10^3^/mm^3^ increase	1.00 (1.00–1.00)	0.04	1.00 (0.99–1.00)	0.08	1.00 (1.00–1.00)	0.7			1.00 (1.00–1.00)	0.3		
Ascites	Yes vs. no	0.85 (0.52–1.41)	0.5			1.68 (1.27–2.23)	< 0.001			1.97 (1.43–2.72)	< 0.001		
Encephalopathy	Yes vs. no	1.31 (0.41–4.12)	0.6			1.79 (0.88–3.65)	0.11			2.40 (1.21–4.76)	0.01		
Esophageal or gastric varices	Yes vs. no	1.52 (0.91–2.54)	0.11			1.71 (1.27–2.31)	< 0.001			1.92 (1.34–2.75)	< 0.001		
Child-Pugh score	1-point increase	0.94 (0.84–1.06)	0.3	1.12 (0.96–1.31)	0.14	1.21 (1.07–1.18)	< 0.001	1.16 (1.09–1.23)	< 0.001	1.21 (1.15–1.28)	< 0.001	1.24 (1.16–1.32)	< 0.001
MELD score	1-point increase	1.01 (0.94–1.07)	0.9			1.05 (1.01–1.08)	0.005			1.06 (1.03–1.10)	< 0.001		

Abbreviations: ALD, alcoholic liver disease; CI, confidence interval; HCC, hepatocellular carcinoma; HCV, hepatitis C virus; NAFLD, non-alcoholic fatty liver disease

In patients with ALD-related cirrhosis, 189 patients discontinued any alcohol intake within the first 12 months following inclusion. Cumulative incidence rates of HCC among abstainers and consumers are presented in S1 Fig. In multivariate analysis among ALD patients, abstinence was not associated with the development of HCC. In patients with HCV-related cirrhosis, 94 received an antiviral therapy and 21 had a sustained virological response following antiviral therapy. Cumulative incidence rates of HCC among patients who achieved viral eradication and those who did not are presented in S2 Fig. In multivariate analysis among HCV patients, viral eradication was not associated with the development of HCC.

### Cumulative incidence of mortality and factors predicting death

**Fig 3:**
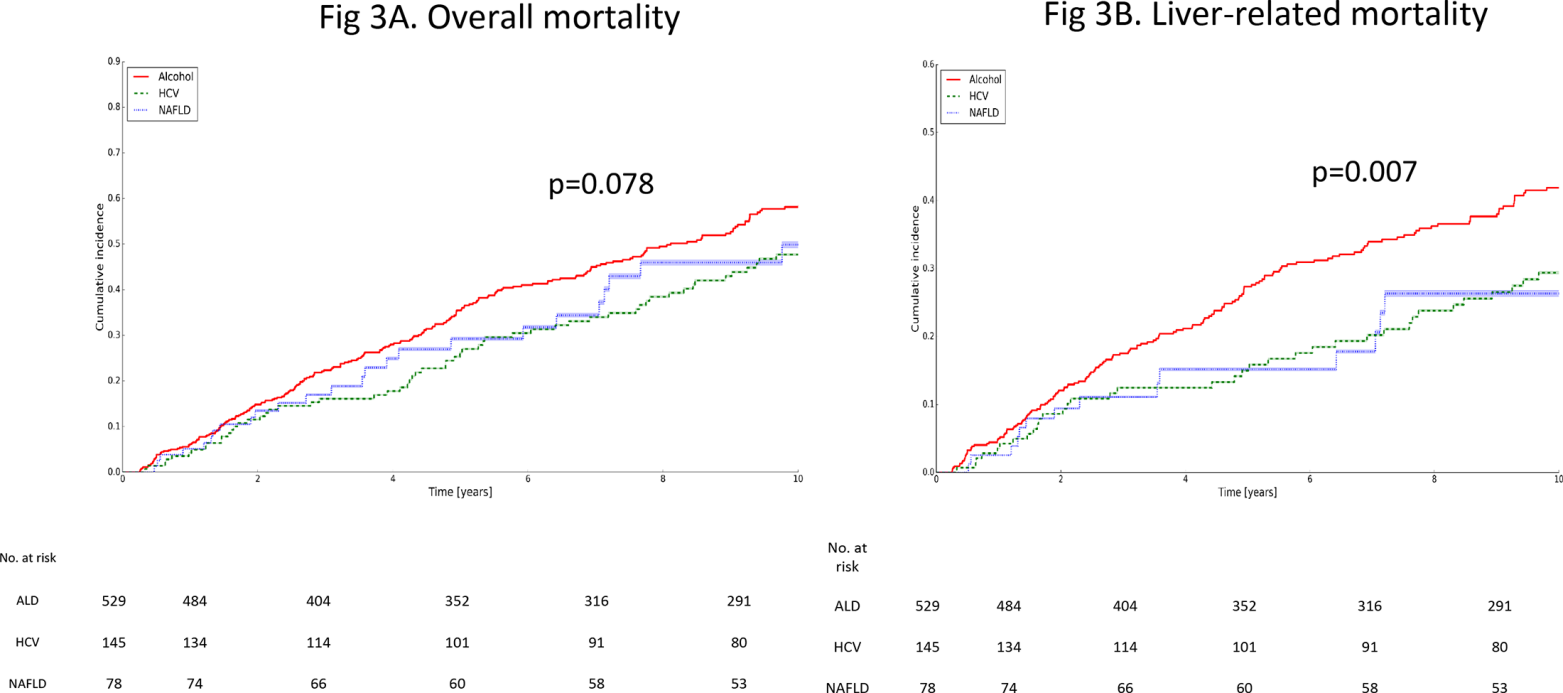
Cumulative incidence rate of mortality according to the cause of cirrhosis. (A). Overall mortality; (B). Liver-related mortality. ALD, alcoholic liver disease; HCV, hepatitis C virus; NAFLD, non-alcoholic fatty liver disease.

S4 Table provides the 5 and 10-year cumulative incidence rates of mortality in patients with ALD, HCV and NAFLD-related cirrhosis. The 10-year cumulative incidence rates of mortality were numerically higher but not significantly different in ALD patients than in HCV or NAFLD patients (58.1% vs. 47.7% vs. 49.9%; p = 0.078, Fig 3A). The annual cumulative incidences of mortality were 8.3%, 7.0% and 7.0% in ALD, HCV and NAFLD patients, respectively.

S5 Table provides the 5 and 10-year cumulative incidence rates of liver-related mortality in patients with ALD, HCV and NAFLD-related cirrhosis. The 10-year cumulative incidence rates of liver-related mortality were significantly higher in ALD patients than in HCV or NAFLD patients (42.0% vs. 29.6% vs. 26.8%, respectively; p = 0.007, Fig 3B). The annual cumulative risks of liver-related mortality were 5.9%, 4% and 3.2% in ALD, HCV and NAFLD patients, respectively.

In time-dependent multivariate proportional hazards models, factors associated with all cause mortality were older age (HR: 1.05; 95% CI, 1.04–1.06; p<0.001), gender (HR for male gender: 1.36; 95% CI, 1.10–1.69; p = 0.005), etiology of cirrhosis (HR for ALD: 1.53; 95% CI, 1.20–1.95; p<0.001), baseline Child-Pugh score (HR: 1.16; 95% CI, 1.09–1.23; p<0.001) (Table 2). Factors associated with liver-related mortality were older age (HR: 1.03; 95% CI, 1.02–1.04; p<0.001), gender (HR for male gender: 1.38; 95% CI, 1.05–1.80; p = 0.02), etiology of cirrhosis (HR for ALD: 1.38; 95% CI, 1.00–1.91; p = 0.05), baseline Child-Pugh score (HR: 1.24; 95% CI, 1.16–1.32; p<0.001) (Table 2). In subgroup analysis restricted to Child-Pugh A patients, factors associated with all-cause mortality were older age (HR: 1.06; 95% CI, 1.04–1.07; p<0.001), etiology of cirrhosis (HR for ALD: 1.90; 95% CI, 1.42–2.53; p<0.001), and baseline Child-Pugh score (HR: 1.77; 95% CI, 1.27–2.47; p<0.001) (S3 Table). Factors associated with liver-related mortality were older age (HR: 1.03; 95% CI, 1.02–1.05; p<0.001), etiology of cirrhosis (HR for ALD: 1.59; 95% CI, 1.08–2.34; p = 0.02), and baseline Child-Pugh score (HR: 1.89; 95% CI, 1.25–2.84; p = 0.002) (S3 Table).

Cumulative incidence rates of death and liver-related death among patients with ALD-related cirrhosis according to abstinence are presented in S3 and S4 Figs. Compared to patients with HCV- or NAFLD-related cirrhosis, abstainers had similar 10-year cumulative incidence rates of mortality (42.2% vs. 47.7% vs. 49.9%, respectively; p = 0.14, S5 Fig) and liver-related mortality (26.9% vs. 29.6% vs. 26.8%, respectively; p = 0.4, S6 Fig). Compared to patients with HCV- or NAFLD-related cirrhosis, consumers had higher 10-year cumulative incidence rates of mortality (63.6% vs. 47.7% vs. 49.9%, respectively; p = 0.003, S7 Fig) and liver-related mortality (46.9% vs. 29.6% vs. 26.8%, respectively; p<0.001, S8 Fig). In multivariate analysis among ALD patients, abstinence was associated with reduced mortality (HR: 0.49; 95% CI, 0.32–0.73; p<0.001) and with reduced liver-related mortality (HR: 0.35; 95% CI, 0.21–0.57; p<0.001). Cumulative incidence rates of death and liver-related death among patients with HCV-related cirrhosis according to viral eradication are presented in S9 and S10 Figs. In multivariate analysis among HCV patients, viral eradication was associated with reduced mortality (HR: 0.47; 95% CI, 0.24–0.95; p = 0.04) but not with reduced liver-related mortality.

## Discussion

Cirrhosis is a major public health problem that requires precise prognosis assessment [9, 17–19]. Studies focusing on patients with severe chronic liver disease usually consider cirrhosis as a unique disease despite the fact that clinical characteristics and outcomes differ according to the etiology of the underlying liver disease. This current study compared the outcomes among 752 patients with ALD, HCV or NAFLD-related cirrhosis during a long period of time. Two main conclusions can be drawn.

The most important finding is that ALD was independently associated with a worse prognosis than HCV infection or NAFLD. Although cumulative incidences of overall mortality were not statistically different between patients, multivariate analysis identified ALD as an independent risk factor for mortality. This result, that may seem surprising, is due to complex and opposite relationship between prognostic factors and survival. If related factors have opposite effects on survival, the univariate effect of each factor is highly confounded [20]. In this analysis, ALD patients were younger than HCV or NAFLD patients, so that, in univariate analysis, the positive survival effect of younger age was confounded by the negative effect of ALD as a cause of cirrhosis, and the univariate test was not significant. However, this conclusion was drawn from the overall analysis which included ALD patients regardless their alcohol intake during follow-up. Large differences were observed in the cumulative incidence rates of mortality between abstainers and consumers. When separating ALD patients according to their alcohol intake, abstainers had similar mortality rates to those of HCV and NAFLD patients. Thus, the poorer prognosis of ALD patients only concerns active consumers. In line with this comment, viral eradication was achieved in a small proportion of HCV patients and was also associated with a reduced mortality. Considering recent progress being made in viral eradication in HCV patients, the prognosis of patients with HCV-related cirrhosis will likely improve in the coming years. By contrast, no etiological treatment of cirrhosis was available for NAFLD patients. Unfortunately, it was not possible to include abstinence or viral eradication in the multivariate analysis as these data only concerned ALD or HCV patients.

The negative impact of ALD on survival of cirrhotic patients has already been observed in one study [21] but not in some other studies [3, 4]. In the first study, a multivariate analysis was not performed. In the latter 2 studies, the lack of impact of ALD on patient prognosis could be explained by a short follow-up period or a limited number of patients. Our study overcomes these limitations by including a large number of patients followed over a 21-year period and by using accurate statistical methodology for assessing patient prognosis, as recommended [11]. The reasons why ALD-related cirrhosis is associated with higher mortality are manifold. First of all, ALD patients had a more severe liver disease at initial presentation, a feature associated with higher mortality rates [22]. In addition, severe acute alcoholic hepatitis, a complication encountered in up to 30% of ALD patients, is associated with a high short-term mortality [23]. Lastly, heavy alcohol intake may worsen the prognosis of serious infections by inducing inappropriate immune response and impaired T lymphocyte cell function [24].

It is often stated that HCC is the main cause of death in patients with cirrhosis [25]. This is clearly not the case in patients with cirrhosis related to ALD. In this study, only 2.3% of patients with ALD-related cirrhosis died from HCC. This is explained by the lower risk of HCC in patients with cirrhosis related to ALD than in those with cirrhosis related to chronic HCV infection or NAFLD. If the incidence of HCC among ALD patients may appear low compared to other studies [26], a similar incidence has been reported in others [3, 4]. Of note, the lower incidence of HCC among ALD patients does not seem to be related to a lack of compliance in HCC screening as the percentage of HCC diagnosed during surveillance with Doppler ultrasonography examination performed every 6 months or within the Milan criteria did not differ compared to the one observed in HCV and NAFLD patients. Even if we cannot exclude the possibility that HCC could have developed in some consumers in whom only data related to death and causes of death were available, this seems unlikely. In addition, the proportion of patients at risk of developing HCC during follow-up did not differ significantly in ALD patients compared to HCV and NAFLD patients. Several lines of evidence indicate that ALD patients have a reduced risk of HCC compared to HCV or NAFLD patients. Firstly, ALD patients were 10 years younger than NAFLD or HCV patients at inclusion, and age is a well-known risk factor for HCC. Hence, the extent of reduction in the risk of HCC observed among ALD patients may be more related to their younger age than to the underlying liver disease itself. Secondly, HCC may complicate HCV infection and NAFLD before cirrhosis occurs, which indicates that cirrhosis is not the only risk factor for HCC in these patients [27]. Thirdly, diabetes mellitus and insulin resistance are frequently encountered in HCV or NAFLD patients and are associated with a higher risk of HCC [28]. Lastly, HCV has direct oncogenic properties [29]. Considering the low incidence of HCC in ALD patients and the fact that nearly half of these patients presented decompensated cirrhosis precluding the feasibility of a curative treatment for HCC, the impact of HCC surveillance on mortality remains to be established in ALD patients.

We acknowledge that our study has several limitations. Firstly, some patients may have been misclassified. This may be particularly relevant for patients with cirrhosis related to NAFLD. However, causes of cirrhosis were determined using a set of clinical, biological, serological and histological data. Most of these data were confirmed on several occasions during the follow-up period. Thus, it is unlikely that the main cause of cirrhosis was wrongly identified. Secondly, data related to the presence of esophageal varices, MELD score, BMI or smoking habits were not available in all patients. When the registry began, MELD score was not yet used as the main criteria for organ donor allocation. However, based on the current literature, MELD score does not perform better than Child-Pugh score in non-transplant settings for predicting the prognosis of patients with cirrhosis [30]. Thirdly, the management of patients with cirrhosis has significantly changed during the study period. On the other hand, all patients were followed by the same physician according to existing practice guidelines regardless of the etiology of cirrhosis. In addition, the year of inclusion was associated neither with overall mortality nor with liver-related mortality (data not shown). Fourthly, few patients underwent a liver transplantation, which may be explained by the low numbers of donors in Belgium, the lack of abstinence and the presence of other comorbidities among ALD patients, or the age of HCV patients when HCC occurred. Fifthly, this study had no external validation cohort. Hence, the conclusion of our study cannot be applied to other centers in which the epidemiology of liver disease differs and our findings should be confirmed in further studies. Finally, in this study designed to assess patients’ prognosis in real life settings, we cannot exclude the existence of a lead-time bias leading to earlier diagnosis of cirrhosis in patients with rapid disease progression. However, a significant impact of a lead-time bias on our results seems unlikely when considering that the median age at death was 15 years lower in ALD patients compared to other patients. Conversely, our study has several robust strengths. First, since patients were included in a single center, at the out-patient clinic by a sole investigator, it could be expected that the inclusion process was exhaustive, homogeneous and rigorous according to well defined criteria. In addition to the long follow-up period and the high number of patients included, only 14 of them were lost to follow-up. As a result, detailed analysis of the causes of death was performed, enabling us to study all causes of mortality as a single outcome as well as liver-related mortality using cumulative incidence functions, as recommended [11].

In conclusion, patients with cirrhosis related to ALD have a lower incidence of HCC but die more frequently from decompensation of cirrhosis than patients with cirrhosis related to chronic HCV infection or NAFLD. Cirrhosis related to ALD should be considered as a condition associated with a poor outcome. This population should deserve specific patient care focused on the management of complications related to liver failure. The impact of HCC surveillance on mortality remains to be established in ALD patients.

## Supporting information

S1 Fig Cumulative incidence rate of HCC among ALD patients according to abstinence. ALD, alcoholic liver disease; HCC, hepatocellular carcinoma.
(TIFF)

S2 Fig Cumulative incidence rate of HCC among HCV patients who achieved viral eradication and those who did not. HCC, hepatocellular carcinoma; HCV, hepatitis C virus.
(TIFF)

S3 Fig Cumulative incidence rate of death among ALD patients according to abstinence. ALD, alcoholic liver disease.
(TIFF)

S4 Fig Cumulative incidence rate of liver-related death among ALD patients according to abstinence. ALD, alcoholic liver disease.
(TIFF)

S5 Fig Cumulative incidence rate of death among ALD patients who abstained from alcohol compared to patients with HCV- or NAFLD-related cirrhosis. ALD, alcoholic liver disease; HCV, hepatitis C virus; NAFLD, non-alcoholic fatty liver disease.
(TIFF)

S6 Fig Cumulative incidence rate of death among ALD patients who did not abstain from alcohol compared to patients with HCV- or NAFLD-related cirrhosis. ALD, alcoholic liver disease; HCV, hepatitis C virus; NAFLD, non-alcoholic fatty liver disease.
(TIFF)

S7 Fig Cumulative incidence rate of liver-related death among ALD patients who abstained from alcohol compared to patients with HCV- or NAFLD-related cirrhosis. ALD, alcoholic liver disease; HCV, hepatitis C virus; NAFLD, non-alcoholic fatty liver disease.
(TIFF)

S8 Fig Cumulative incidence rate of liver-related death among ALD patients who did not abstain from alcohol compared to patients with HCV or NAFLD-related cirrhosis. ALD, alcoholic liver disease; HCV, hepatitis C virus; NAFLD, non-alcoholic fatty liver disease.
(TIFF)

S9 Fig Cumulative incidence rate of death among HCV patients who achieved viral eradication and those who did not. HCC, hepatocellular carcinoma; HCV, hepatitis C virus.
(TIFF)

S10 Fig Cumulative incidence rate of liver-related death among HCV patients who achieved viral eradication and those who did not. HCC, hepatocellular carcinoma; HCV, hepatitis C virus.
(TIFF)

S1 Table Clinical events according to the cause of cirrhosis. ALD, alcoholic liver disease; CI, confidence interval; HCC, hepatocellular carcinoma; HCV, hepatitis C virus; NAFLD, non-alcoholic fatty liver disease.
(DOCX)

S2 Table 5 and 10-year cumulative incidence rates of HCC in patients with ALD, HCV and NAFLD-related cirrhosis. ALD, alcoholic liver disease; HCC, hepatocellular carcinoma; HCV, hepatitis C virus; NAFLD, non-alcoholic fatty liver disease.
(DOCX)

S3 Table Risk Factors for HCC and for death in subgroup analysis restricted to Child-Pugh A patients. ALD, alcoholic liver disease; CI, confidence interval; HCC, hepatocellular carcinoma; HCV, hepatitis C virus; NA, not available (as no patients with encephalopathy developed HCC in this subgroup analysis); NAFLD, non-alcoholic fatty liver disease.
(DOCX)

S4 Table 5 and 10-year cumulative incidence rates of mortality in patients with ALD, HCV and NAFLD-related cirrhosis. ALD, alcoholic liver disease; HCV, hepatitis C virus; NAFLD, non-alcoholic fatty liver disease.
(DOCX)

S5 Table 5 and 10-year cumulative incidence rates of liver-related mortality in patients with ALD, HCV and NAFLD-related cirrhosis. ALD, alcoholic liver disease; HCV, hepatitis C virus; NAFLD, non-alcoholic fatty liver disease.
(DOCX)

## Abbreviations

AIC: Akaike information criterion
ALD: alcoholic liver disease
BMI: body mass index
CI: confidence interval
HBV: hepatitis B virus
HCC: hepatocellular carcinoma
HCV: hepatitis C virus
HR: hazard ratio
MELD: model for end-stage liver disease
NAFLD: nonalcoholic fatty liver disease
NASH: nonalcoholic steatohepatitis
SVR: Sustained virological response
