# Supplementary figures and images for: Alcoholic liver disease confers a worse prognosis than HCV infection and non-alcoholic fatty liver disease among patients with cirrhosis: An observational study
Source: PLoS One. 2017 Oct 27;12(10):e0186715. doi: 10.1371/journal.pone.0186715 (PMC5659599; doi:10.1371/journal.pone.0186715)

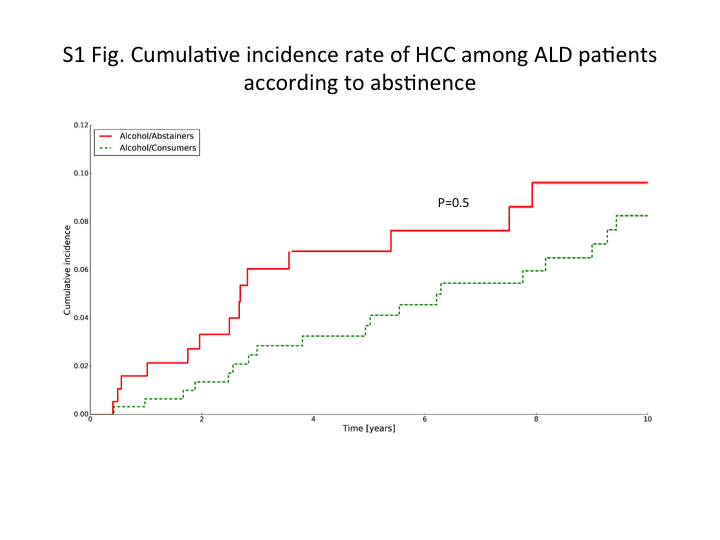

Supplement: S1 Fig — ALD, alcoholic liver disease; HCC, hepatocellular carcinoma. (TIFF) [file pone.0186715.s001.tiff]

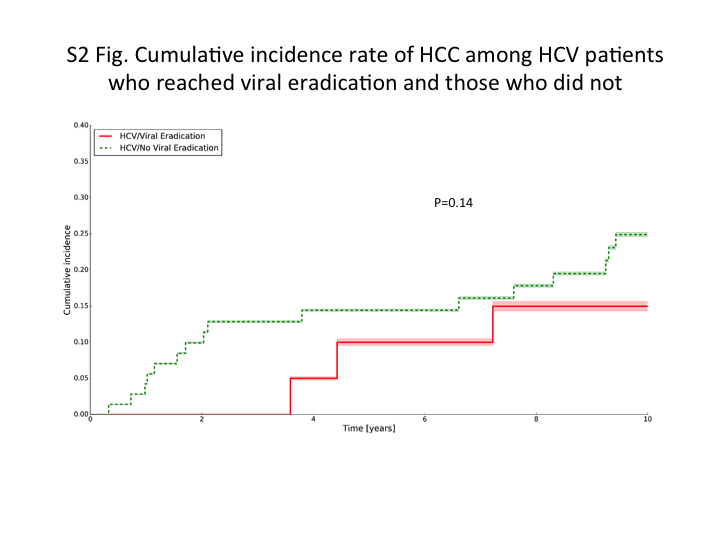

Supplement: S2 Fig — HCC, hepatocellular carcinoma; HCV, hepatitis C virus. (TIFF) [file pone.0186715.s002.tiff]

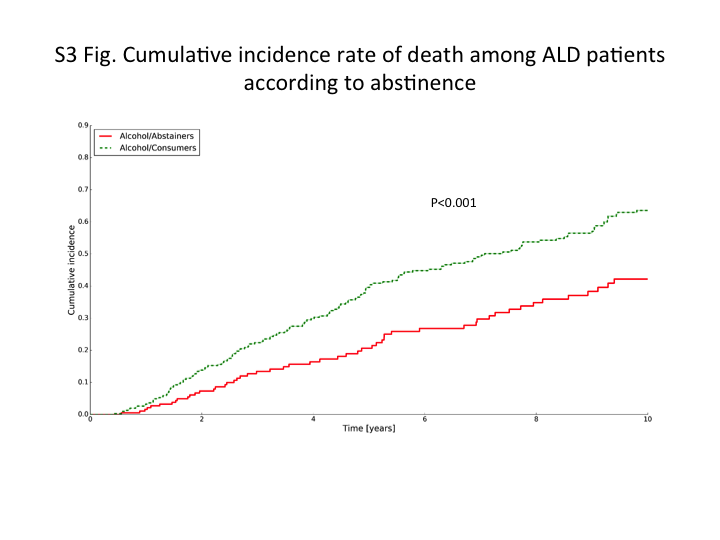

Supplement: S3 Fig — ALD, alcoholic liver disease. (TIFF) [file pone.0186715.s003.tiff]

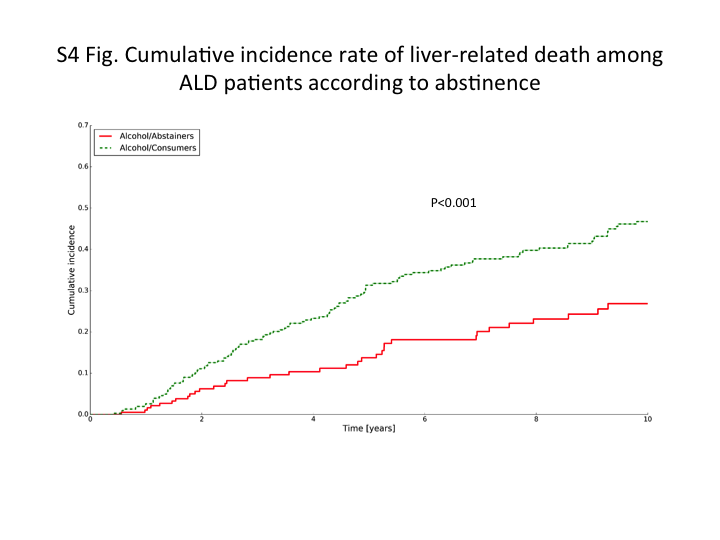

Supplement: S4 Fig — ALD, alcoholic liver disease. (TIFF) [file pone.0186715.s004.tiff]

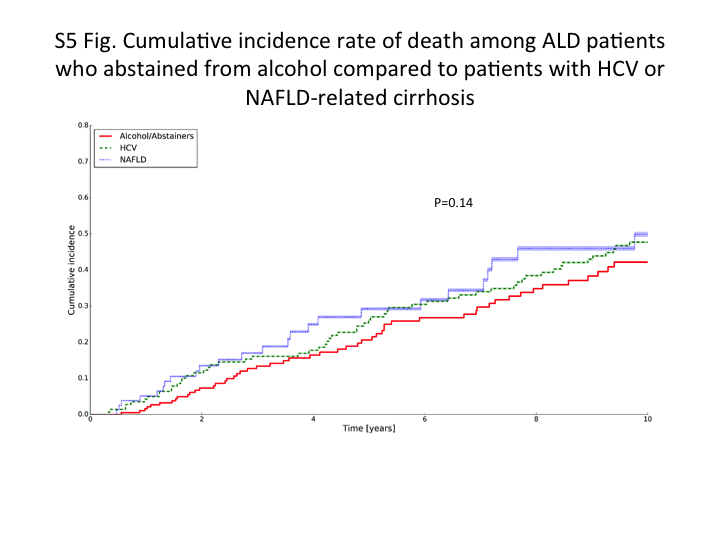

Supplement: S5 Fig — ALD, alcoholic liver disease; HCV, hepatitis C virus; NAFLD, non-alcoholic fatty liver disease. (TIFF) [file pone.0186715.s005.tiff]

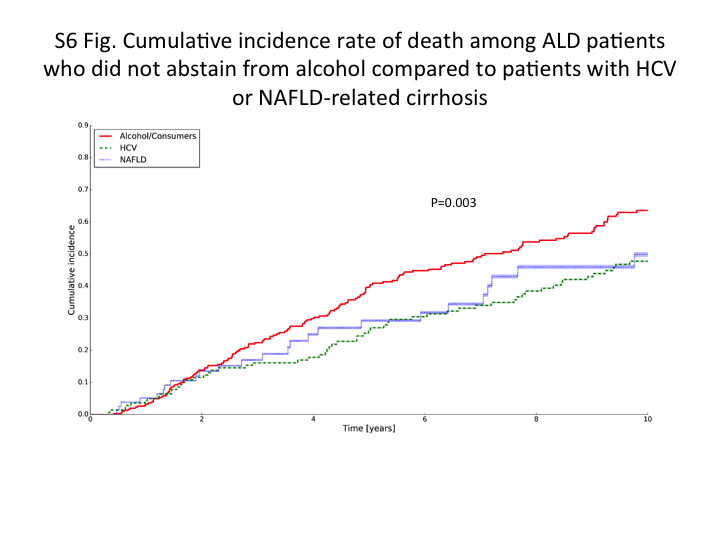

Supplement: S6 Fig — ALD, alcoholic liver disease; HCV, hepatitis C virus; NAFLD, non-alcoholic fatty liver disease. (TIFF) [file pone.0186715.s006.tiff]

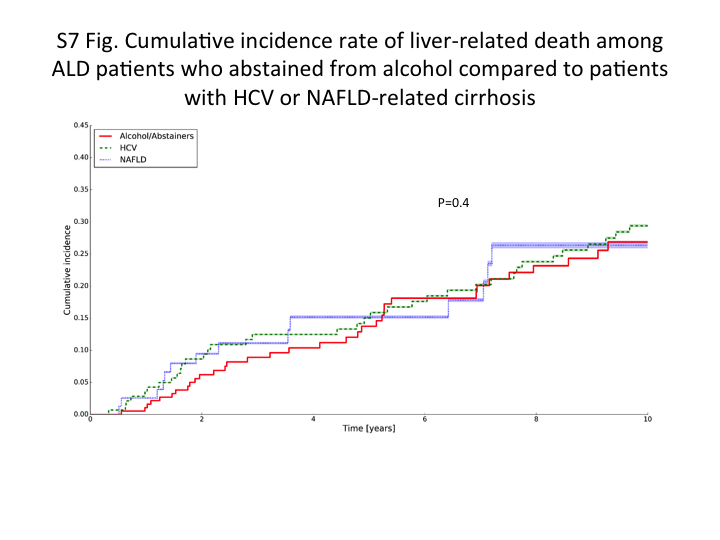

Supplement: S7 Fig — ALD, alcoholic liver disease; HCV, hepatitis C virus; NAFLD, non-alcoholic fatty liver disease. (TIFF) [file pone.0186715.s007.tiff]

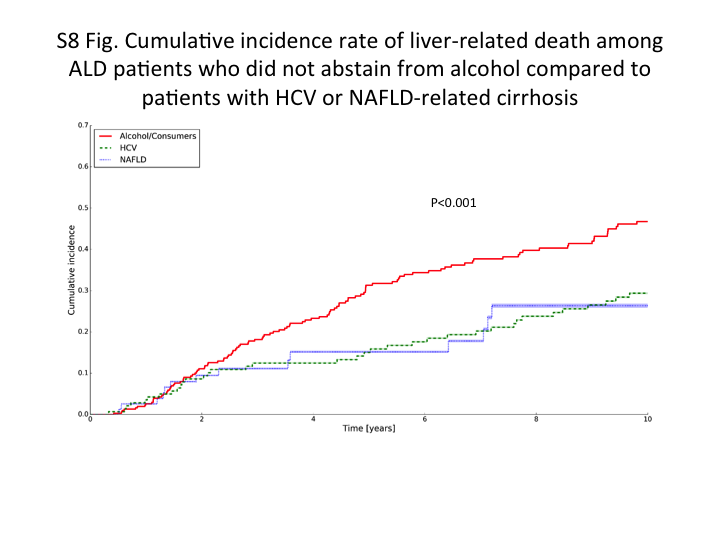

Supplement: S8 Fig — ALD, alcoholic liver disease; HCV, hepatitis C virus; NAFLD, non-alcoholic fatty liver disease. (TIFF) [file pone.0186715.s008.tiff]

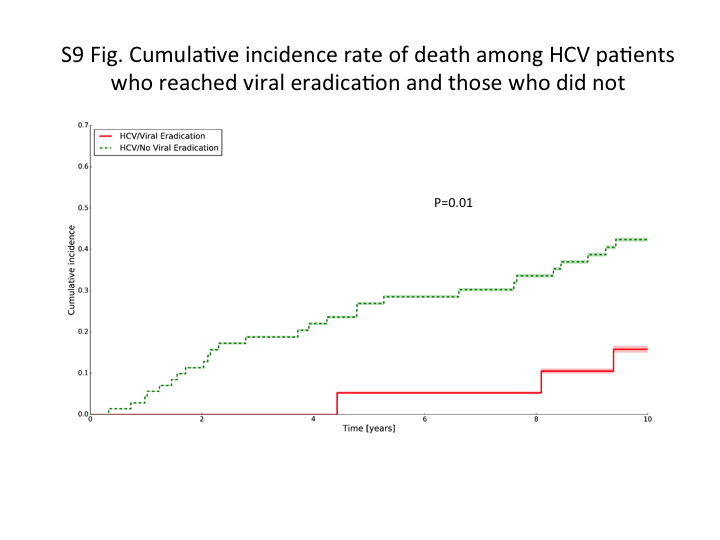

Supplement: S9 Fig — HCC, hepatocellular carcinoma; HCV, hepatitis C virus. (TIFF) [file pone.0186715.s009.tiff]

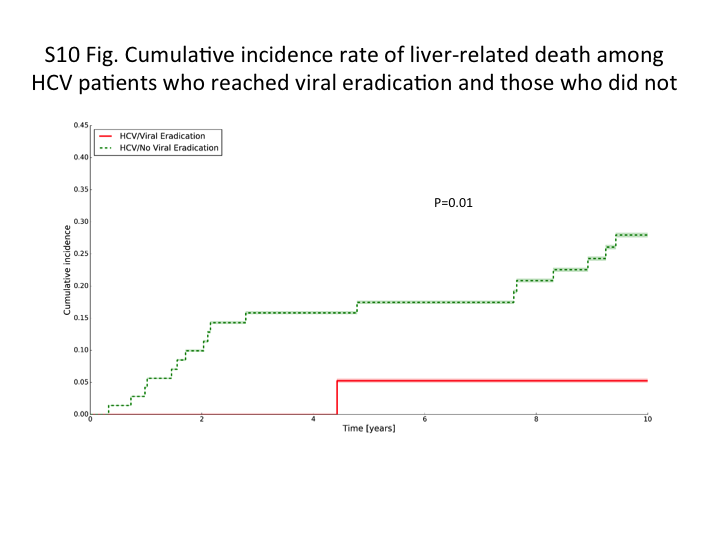

Supplement: S10 Fig — HCC, hepatocellular carcinoma; HCV, hepatitis C virus. (TIFF) [file pone.0186715.s010.tiff]
