# Supplementary material for: Alcoholic liver disease confers a worse prognosis than HCV infection and non-alcoholic fatty liver disease among patients with cirrhosis: An observational study
Source: PLoS One. 2017 Oct 27;12(10):e0186715. doi: 10.1371/journal.pone.0186715 (PMC5659599; doi:10.1371/journal.pone.0186715)
Supplement: S1 Table — ALD, alcoholic liver disease; CI, confidence interval; HCC, hepatocellular carcinoma; HCV, hepatitis C virus; NAFLD, non-alcoholic fatty liver disease. (DOCX) [file pone.0186715.s011.docx]

**S1 Table. Clinical events according to the cause of cirrhosis.**

| **Characteristics*** | **Whole population**  **(n=752)** | **ALD group**  **(n=529)** | **HCV group**  **(n=145)** | **NAFLD group**  **(n=78)** | ***p-Value*** |
| --- | --- | --- | --- | --- | --- |
| HCC (no., %) | 85 (11%) | 38 (7%) | 35 (24%) | 12 (15%) | < 0.001 |
| Deaths (no., %) * | 379 (51%) | 273 (53%) | 78 (55%) | 28 (36%) | 0.01 |
| Liver-related deaths (no., % of deaths) | 250 (66%) | 190 (70%) | 46 (59%) | 14 (50%) | <0.001 |
| HCC-related deaths (no., % of deaths) | 37 (10%) | 12 (5%) | 21 (27%) | 4 (14%) |  |
| Non-HCC liver-related deaths (no., % of deaths) | 213 (56%) | 178 (65%) | 25 (32%) | 10 (36%) |  |
| End-stage liver disease (no., % of deaths) | 93 (24%) | 75 (27%) | 13 (17%) | 5 (18%) |  |
| Acute-on-chronic liver failure (no., % of deaths) | 120 (32%) | 103 (38%) | 12 (15%) | 5 (18%) |  |
| Gastrointestinal bleeding (no., % of deaths) | 41 (11%) | 35 (13%) | 5 (6%) | 1 (4%) |  |
| Alcoholic hepatitis (no., % of deaths) | 29 (8%) | 29 (11%) | 0 (0%) | 0 (0%) |  |
| Sepsis (no., % of deaths) | 25 (6.5%) | 20 (7%) | 2 (3%) | 3 (10%) |  |
| Others (no., % of deaths) | 25 (6.5%) | 19 (7%) | 5 (6%) | 1 (4%) |  |
| Non liver-related deaths (no., % of deaths) | 123 (32%) | 80 (29%) | 30 (38%) | 13 (46%) | < 0.001 |
| Death from unknown cause (no., % of deaths) | 6 (2%) | 3 (1%) | 2 (3%) | 1 (4%) | < 0.001 |
| Liver transplantation (no., %) * | 27 (4%) | 17 (3%) | 8 (5%) | 2 (3%) | 0.4 |

Abbreviations: ALD, alcoholic liver disease; HCC, hepatocellular carcinoma; HCV, hepatitis C virus; NAFLD, non-alcoholic fatty liver disease

* Data available in 738 patients
