# Supplementary material for: Alcoholic liver disease confers a worse prognosis than HCV infection and non-alcoholic fatty liver disease among patients with cirrhosis: An observational study
Source: PLoS One. 2017 Oct 27;12(10):e0186715. doi: 10.1371/journal.pone.0186715 (PMC5659599; doi:10.1371/journal.pone.0186715)
Supplement: S2 Table — ALD, alcoholic liver disease; HCC, hepatocellular carcinoma; HCV, hepatitis C virus; NAFLD, non-alcoholic fatty liver disease. (DOCX) [file pone.0186715.s012.docx]

**S2 Table. 5 and 10-year cumulative incidence rates of HCC in patients with ALD, HCV and NAFLD-related cirrhosis.**

| **Characteristics** | **Whole study population** | ***p-Value*** |
| --- | --- | --- |
| **Patients with ALD-related cirrhosis** |  | < 0.001 |
| 5-year cumulative incidence rate of HCC (95% CI) | 4.6% (2.7 – 6.5) |  |
| 10-year cumulative incidence rate of HCC (95% CI) | 8.4% (5.6 – 11.2) |  |
| **Patients with HCV-related cirrhosis** |  |  |
| 5-year cumulative incidence rate of HCC (95% CI) | 10.5% (5.2 – 15.8) |  |
| 10-year cumulative incidence rate of HCC (95% CI) | 22.0% (14.6 – 29.4) |  |
| **Patients with NAFLD-related cirrhosis** |  |  |
| 5-year cumulative incidence rate of HCC (95% CI) | 11.2% (3.1 – 19.3) |  |
| 10-year cumulative incidence rate of HCC (95% CI) | 23.7% (9.9 – 37.5) |  |

Abbreviations: ALD, alcoholic liver disease; CI, confidence interval; HCC, hepatocellular carcinoma; HCV, hepatitis C virus; NAFLD, non-alcoholic fatty liver disease
