# Supplementary material for: Alcoholic liver disease confers a worse prognosis than HCV infection and non-alcoholic fatty liver disease among patients with cirrhosis: An observational study
Source: PLoS One. 2017 Oct 27;12(10):e0186715. doi: 10.1371/journal.pone.0186715 (PMC5659599; doi:10.1371/journal.pone.0186715)
Supplement: S3 Table — ALD, alcoholic liver disease; CI, confidence interval; HCC, hepatocellular carcinoma; HCV, hepatitis C virus; NA, not available (as no patients with encephalopathy developed HCC in this subgroup analysis); NAFLD, non-alcoholic fatty liver disease. (DOCX) [file pone.0186715.s013.docx]

**S3 Table. Risk Factors for HCC and for death in subgroup analysis restricted to Child-Pugh A patients.**

|  |  | **HCC** | | | | **Mortality** | | | | **Liver-related mortality** | | | |
| --- | --- | --- | --- | --- | --- | --- | --- | --- | --- | --- | --- | --- | --- |
|  |  | **Univariate** | | **Multivariate** | | **Univariate** | | **Multivariate** | | **Univariate** | | **Multivariate** | |
| **Baseline characteristics** | **Comparison group** | **Hazard ratio**  **(95% CI)** | ***p-Value*** | **Hazard ratio**  **(95% CI)** | ***p-Value*** | **Hazard ratio**  **(95% CI)** | ***p-Value*** | **Hazard ratio**  **(95% CI)** | ***p-Value*** | **Hazard ratio**  **(95% CI)** | ***p-Value*** | **Hazard ratio**  **(95% CI)** | ***p-Value*** |
| Age | 1-year increase | 1.04 (1.02 – 1.06) | < 0.001 | 1.02 (0.98-1.05) | 0.3 | 1.04 (1.03-1.05) | < 0.001 | 1.06 (1.04-1.07) | < 0.001 | 1.02 (1.01-1.04) | 0.01 | 1.03 (1.02-1.05) | < 0.001 |
| Gender | Male vs. female | 1.95 (1.06 – 3.56) | 0.03 | 3.57 (1.83-6.96) | <0.001 | 1.10 (0.83 – 1.46) | 0.5 | 1.12 (0.85 – 1.48) | 0.4 | 1.11 (0.77 – 1.61) | 0.6 | 1.13 (0.77 – 1.68) | 0.5 |
| Etiology of cirrhosis | ALD vs. non ALD | 0.27 (0.15 – 0.48) | < 0.001 | 0.37 (0.18-0.77) | 0.008 | 1.29 (0.98 – 1.68) | 0.067 | 1.90 (1.42 – 2.53) | < 0.001 | 1.33 (0.93 – 1.92) | 0.12 | 1.59 (1.08 – 2.34) | 0.02 |
| Diabetes | Yes vs. no | 1.69 (0.95 – 3.01) | 0.07 | 1.77 (1.00-3.15) | 0.05 | 1.11 (0.75 – 1.64) | 0.6 |  |  | 0.96 (0.59 – 1.56) | 0.9 |  |  |
| Tobacco use | Yes vs. no | 1.79 (0.72 – 4.44) | 0.2 |  |  | 0.79 (0.47 – 1.32) | 0.4 |  |  | 0.57 (0.29 – 1.11) | 0.1 |  |  |
| BMI | 1-point increase | 1.00 (0.94 – 1.07) | 1.0 |  |  | 0.98 (0.94 – 1.02) | 0.3 |  |  | 0.97 (0.92 – 1.03) | 0.4 |  |  |
| Bilirubin | 1 mg/dl increase | 0.74 (0.40 – 1.40) | 0.4 |  |  | 1.29 (0.92 – 1.81) | 0.14 |  |  | 1.42 (0.97 – 2.09) | 0.071 |  |  |
| INR | 1-point increase | 0.05 (0.00 – 2.96) | 0.15 |  |  | 1.56 (0.25 – 9.83) | 0.6 |  |  | 1.76 (0.19 – 16.55) | 0.6 |  |  |
| Albumin | 1 g/dl increase | 1.13 (0.60 – 2.12) | 0.7 |  |  | 0.65 (0.45 – 0.94) | 0.024 |  |  | 0.78 (0.52 – 1.17) | 0.2 |  |  |
| Creatinin | 1 mg/dl increase | 4.12 (1.22 – 13.93) | 0.02 |  |  | 3.02 (1.02 – 8.95) | 0.047 |  |  | 2.86 (0.73 – 11.21) | 0.13 |  |  |
| Platelet count | 10^3^/mm³ increase | 0.99 (0.99 – 1.00) | 0.014 |  |  | 1.00 (1.00 – 1.00) | 0.9 |  |  | 1.00 (0.99 – 1.00) | 0.4 |  |  |
| Ascites | Yes vs. no | 0.51 (0.12 – 2.17) | 0.4 |  |  | 1.44 (0.79 – 2.62) | 0.2 |  |  | 1.36 (0.63 – 2.93) | 0.4 |  |  |
| Encephalopathy | Yes vs. no | NA | NA |  |  | 8.83 (6.63 – 11.75) | < 0.001 |  |  | 12.01 (8.62 – 16.72) | <0.001 |  |  |
| Esophageal or gastric varices | Yes vs. no | 1.19 (0.63 – 2.24) | 0.6 |  |  | 2.03 (1.36 – 3.01) | < 0.001 |  |  | 2.06 (1.25 – 3.38) | 0.004 |  |  |
| Child-Pugh score | 1-point increase | 0.75 (0.37 – 1.54) | 0.4 | 0.71 (0.31 – 1.65) | 0.4 | 1.61 (1.18 – 2.20) | 0.03 | 1.77 (1.27-2.47) | < 0.001 | 1.84 (1.24 – 2.75) | 0.003 | 1.89 (1.25 – 2.84) | 0.002 |
| MELD score | 1-point increase | 0.96 (0.82 – 1.11) | 0.6 |  |  | 1.02 (0.97 – 1.07) | 0.5 |  |  | 1.03 (0.98 – 1.08) | 0.3 |  |  |

Abbreviations: ALD, alcoholic liver disease; CI, confidence interval; HCC, hepatocellular carcinoma; HCV, hepatitis C virus; NA, not available (as no patients with encephalopathy developed HCC in this subgroup analysis); NAFLD, non-alcoholic fatty liver disease
