# Supplementary material for: Alcoholic liver disease confers a worse prognosis than HCV infection and non-alcoholic fatty liver disease among patients with cirrhosis: An observational study
Source: PLoS One. 2017 Oct 27;12(10):e0186715. doi: 10.1371/journal.pone.0186715 (PMC5659599; doi:10.1371/journal.pone.0186715)
Supplement: S4 Table — ALD, alcoholic liver disease; HCV, hepatitis C virus; NAFLD, non-alcoholic fatty liver disease. (DOCX) [file pone.0186715.s014.docx]

**S4 Table. 5 and 10-year cumulative incidence rates of mortality in patients with ALD, HCV and NAFLD-related cirrhosis.**

| **Characteristics** | **Whole study population** | ***p-Value*** |
| --- | --- | --- |
| **Patients with ALD-related cirrhosis** |  | 0.078 |
| 5-year cumulative incidence rate of mortality (95% CI) | 35.5% (31.0 – 40.0) |  |
| 10-year cumulative incidence rate of mortality (95% CI) | 58.1% (52.8 – 63.4) |  |
| **Patients with HCV-related cirrhosis** |  |  |
| 5-year cumulative incidence rate of mortality (95% CI) | 26.2% (18.5 – 33.9) |  |
| 10-year cumulative incidence rate of mortality (95% CI) | 47.7% (38.6 – 56.8) |  |
| **Patients with NAFLD-related cirrhosis** |  |  |
| 5-year cumulative incidence rate of mortality (95% CI) | 29.3% (17.3 – 41.3) |  |
| 10-year cumulative incidence rate of mortality (95% CI) | 49.9% (33.9 – 65.9) |  |

Abbreviations: ALD, alcoholic liver disease; CI, confidence interval; HCV, hepatitis C virus; NAFLD, non-alcoholic fatty liver disease
