# Supplementary material for: Alcoholic liver disease confers a worse prognosis than HCV infection and non-alcoholic fatty liver disease among patients with cirrhosis: An observational study
Source: PLoS One. 2017 Oct 27;12(10):e0186715. doi: 10.1371/journal.pone.0186715 (PMC5659599; doi:10.1371/journal.pone.0186715)
Supplement: S5 Table — ALD, alcoholic liver disease; HCV, hepatitis C virus; NAFLD, non-alcoholic fatty liver disease. (DOCX) [file pone.0186715.s015.docx]

**S5 Table: 5 and 10-year cumulative incidence rates of liver-related mortality in patients with ALD, HCV and NAFLD-related cirrhosis**

| **Characteristics** | **Whole study population** | ***p-Value*** |
| --- | --- | --- |
| **Patients with ALD-related cirrhosis** |  | 0.007 |
| 5-year cumulative incidence rate of liver-related mortality (95% CI) | 27.4% (23.2 – 31.6) |  |
| 10-year cumulative incidence rate of liver-related mortality (95% CI) | 42.0% (36.9 – 47.1) |  |
| **Patients with HCV-related cirrhosis** |  |  |
| 5-year cumulative incidence rate of liver-related mortality (95% CI) | 15.0% (8.9 – 21.1) |  |
| 10-year cumulative incidence rate of liver-related mortality (95% CI) | 29.6% (21.3 – 37.9) |  |
| **Patients with NAFLD-related cirrhosis** |  |  |
| 5-year cumulative incidence rate of liver-related mortality (95% CI) | 15.2% (6.2 – 24.2) |  |
| 10-year cumulative incidence rate of liver-related mortality (95% CI) | 26.8% (13.6 – 40.0) |  |

Abbreviations: ALD, alcoholic liver disease; CI, confidence interval; HCV, hepatitis C virus; NAFLD, non-alcoholic fatty liver disease
